# Notes and News

**Published:** 1894-06-09

**Authors:** 


### Notes and News.

Collected and Communicated.

The Birmingham Hospital Saturday Fund has now reached £11,556 12s., an increase of £229 6s. on the collection of last year. The number of contributors this year is not very much larger than last, and there is therefore a good field which might yet be canvassed to subscribe and secure the desired sum of £12,000.

The new infirmary at Derby is to be opened on Saturday, July 7th. The building operations have extended over three years, and much care and thought has been exercised to provide a model institution for the sick poor of Derby.

The British Medical Association will meet this year at Bristol on the four days succeeding July 30th. The President is Dr. George Hare Philipson and the President-Elect Dr. E. Long Fox. The subjects under consideration are classified under ten sections, and many distinguished members of the profession are to read papers and take part in the discussions.

Times are hard enough for the general practitioner; they will be harder still if his chances of redress are gradually curtailed as they seem to be in some directions. Quite recently at Dundee a doctor was called in to attend at a workhouse in the absence of the official medical attendant. On applying for a fee he was refused payment, and on carrying the matter before the Sheriff's Court his case was dismissed, under the plea that, having no contract with the parochial board, he was not entitled to recover. The decision, unfortunately, was not open to appeal.

Mr. C. S. Loch, Secretary of the Charity Organisation Society, read an interesting paper in St. Martin's Town Hall last week on Metropolitan Pauperism. He devoted a good deal of time to show that the statements of various writers advocating outdoor relief against indoor relief were fallacious. He warmly advocated the wisdom of the system which provided indoor relief for the aged and sick in a judicious manner, and affirmed that the tendency of such a system was shown by experience to decrease their dependence on the public, and that therefore the diminution of outdoor relief does not drive the aged poor into the workhouse, as some contest.

The Army Medical Department of Germany is being materially strengthened. Nine posts of surgeon-majors are about to be added to the William Institute of Military Medicine. This addition will give army doctors an opportunity of gaining useful experience and knowledge in various directions.

The Wistar Institute of Anatomy, in connection with the University of Pennsylvania, has been opened. Dr. Harrison Allen has been appointed Director, whilst Dr. Milton Greenman is to occupy the post of Assistant Director. A new medical college is proposed for Kansas. A Bill for providing registration of physicians in Massachusetts has passed the Senate.

An amusing reason for a strike has occurred at a French lucifer manufactory. To these industries a dentist is attached and visits to him are compulsory. At one factory the dentist was so dreaded from the pain he inflicted that the employés went on strike until another dentist was appointed in his place.

A most successful fête and fancy fair has been held at Belfast in aid of the Students' Union, in connection with Queen's College. The festivities lasted for six days, during which time most varied and attractive performances were held. A most popular feature of the proceedings was a grand military spectacle, entiled the Battle of Tel-el-Kebir, given by 150 students under skilled supervision from London.

Mr. Charles Montague, 4, Bedford Square, E., writes to us asking for help towards sending some of the ailing children of King Edward's Ragged Schools, Albert Street, Spitalfields, to the Country Home of the institution or to the sea for a fortnight, and the other children for a day's outing. Last year 500 children were sent away for from one to four weeks, with a beneficial effect hardly to be realised, and some 2,300 enjoyed a day in the country. This is the only taste of summer pleasures these poor little ones get through the long hot days. Will not our readers help towards a fund for so excellent a purpose?

Amongst those selected to receive birthday honours are numbered several names well known in medical circles. Professor Grainger Stewart, M.D. of Edinburgh, has received the order of knighthood. As professor of the practice of medicine in Edinburgh he has had a distinguished career. In 1887 he was elected an honorary M.D. of the Royal University of Ireland, and a Fellow of the Royal College of Physicians of Ireland. Another Knight, the Hon. Arthur Renwick, member of the Legislative Council of New South Wales, who is also an Edinburgh M.D., and a Fellow of the Royal College of Surgeons of Edinburgh Surgeon-General James Mouat, V.C., C.B., has now retired from the Army Medical Department, of which he was a distinguished member. He has been made a Knight Commander of the Bath. Surgeon-General W. Manley, V.C., who has served in the Crimea and New Zealand war, and is distinguished for many gallant deeds connected with the saving of life, has been created a Companion of the Bath. Fleet-Surgeon W. R. White, R.N., Surgeon W. Bowden, R.N., and Dr. Robert Grieve, Surgeon-General of the Colony of British Guiana, have also received marks of distinction for good service.

